# Identification of potential welfare and survival indicators for stranded cetaceans through international, interdisciplinary expert opinion

**DOI:** 10.1098/rsos.220646

**Published:** 2022-10-12

**Authors:** Rebecca M. Boys, Ngaio J. Beausoleil, Matthew D. M. Pawley, Katherine E. Littlewood, Emma L. Betty, Karen A. Stockin

**Affiliations:** ^1^ Cetacean Ecology Research Group, School of Natural Sciences, College of Sciences, Massey University, Private Bag 102-904, Auckland, New Zealand; ^2^ School of Mathematical and Computational Sciences, College of Sciences, Massey University, Private Bag 102-904, Auckland, New Zealand; ^3^ Animal Welfare Science and Bioethics Centre, School of Veterinary Science, College of Sciences, Massey University, Private Bag 11-222, Palmerston North, New Zealand

**Keywords:** animal welfare, Delphi, management, marine mammals, strandings, wildlife

## Abstract

Management of live cetacean strandings generally focuses on refloating animals, yet there is a lack of scientific data to inform decision-making. Valid indicators that are practical to measure are needed to assess welfare status and survival likelihood for stranded cetaceans. The Delphi method was applied to gather international and interdisciplinary expert opinion to provide face validity to potential indicators of stranded cetacean welfare and survival likelihood. Two online questionnaires were conducted. In the first questionnaire these experts identified potential indicators of stranded cetacean welfare and survival likelihood. These indicators were subsequently scored by the same experts in questionnaire two, based on their value for assessing welfare/survival likelihood and being practical to measure. Indicators considered valuable and practical for assessing welfare and survival likelihood at strandings included animal-based indices of body and skin condition, signs of physical trauma, respiration rate and various behaviours. Resource-/management-based indicators related mainly to human intervention and should be correlated with animal-based indices to provide relevant evaluations. Importantly, inextricable links between welfare and survival for stranded cetaceans are emphasized, with 90% of indicators being similar for both. Investigations into these indicators should be conducted to develop a practical, science-based assessment framework to inform decision-making during stranding events.

## 1. Introduction

There is increasing recognition that animal welfare science must be integrated alongside conservation biology to achieve wildlife management goals [1–4]. While conservation efforts involving human intervention often claim to consider animal welfare, robust welfare assessments are rarely undertaken [5–9]. This is probably due to limited data and protocols for assessing the welfare of wild species [10], and the need to apply practical and non-invasive indicators [11,12]. Likewise, the current paucity of validated prognostic indicators [13] and limited survivorship data for refloated stranded cetaceans [14–16] hinders current ability to undertake informed decisions based on an animal's predicted likelihood to survive. Notably, inextricable links between welfare and survival of stranded cetaceans, and the need to assess both concepts to inform decision-making at stranding events, has been highlighted [17].

In contemporary animal welfare science, it is generally understood that physical and mental states are linked and that an animal's welfare state is based on how it is experiencing its own life [1,18]. Therefore, to be considered important for animal welfare, a physical state or external condition must impact upon the animal's overall subjective mental state [19,20]. Thus, animal welfare is only considered for sentient species, which include, among others, cetaceans [21–23], that are able to experience both negative and positive subjective mental experiences depending on their circumstances [18,19,24].

Since subjective mental experiences cannot be measured directly, they must be inferred through scientific evaluation of an animal's physical state and their external conditions [1,20,25]. To ensure a systematic approach towards evaluations, assessment frameworks, such as the Five Domains Model, are commonly applied [26,27]. Using such a framework, indicators related to physical/functional domains (nutrition, physical environment and health) and the situation-related (external conditions) domain (behavioural interactions) are observed and/or measured. The cumulative evaluation of these indicators are subsequently used to cautiously infer the animal's potential mental (affective) experiences (fifth domain) that they probably reflect [1,28,29].

To apply this kind of systematic framework, measurable and/or observable indicators must first be identified and validated. Indicators can be animal-based (e.g. body condition or specific behaviours) or may be resource- or management-based (e.g. environmental conditions, human interventions). Animal-based indicators provide more direct evidence of the animal's welfare state than resource-/management-based indicators. Welfare indicators can be further categorized into ‘welfare status’ or ‘welfare alerting’. Welfare status indicators provide explicit evidence of an animal's physical state or external situation and therefore more directly reflect its welfare status (i.e. subjective mental experience) [10]. They include some animal-based indicators (e.g. external injuries, specific behaviours). By contrast, welfare alerting indicators do not provide information directly related to an animal's welfare state, but rather represent factors that might compromise that state in some animals exposed to those conditions (i.e. they represent a welfare risk) [10]. These include some animal-based indicators (e.g. age class, reproductive status), but also include all resource-/management-based indicators. Importantly, this kind of contextual information regarding the animal's situation at the time of assessment is always required to appropriately interpret, and give valence to, animal-based indicators [12,30].

Likewise, knowledge of, and the ability to apply, prognostic indicators relevant to an animals' survival likelihood are key to undertaking appropriate, informed management decisions. In the case of stranded cetaceans, indicators relevant to survival likelihood should reflect aspects of an animals’ biological functioning, behaviour and a minimum of 6 months survival post-refloating [17].

Globally, cetacean stranding events appear to have increased in frequency in recent decades [31–35]; a trend which is likely to continue due to climate change [36] and human disturbance [37]. Live strandings often involve costly and complex human intervention. Stranding response aims to achieve conservation goals through reflotation of individual animals, and should also assess health and provide supportive care [38–40]. Stranded cetaceans deemed to be viable may be refloated, or transported and then refloated, while those in poor condition may be taken into rehabilitation centres (where legal and appropriate facilities exist) [41] or end-of-life decisions, such as euthanasia or palliative care, may be required [5,38,42]. While intervention decisions should be informed by the status of the animal, based on both its welfare and its survival likelihood [17,43], undertaking these assessments can be complex. Indeed, limited empirical data exist to support decision-making at strandings [13,16,44–46], which is considered a potential major cetacean welfare concern [47].

Since empirical data is lacking, a preliminary way to acquire information on potential indicators is to elicit expert opinion [48–50]. One way this can be achieved is via the application of the Delphi method [51], which assumes that group opinion is more representative than individual opinion [52]. This method has previously identified and validated indicators of animal welfare state in varying animal species [53–56]. With regard to management of live cetacean strandings, expert opinion can provide face validity to indicators (the extent to which indicators align with welfare state/survival likelihood) relevant to developing a framework [57] for assessing stranded cetacean welfare and survival likelihood. These data can subsequently be applied at live cetacean stranding events to investigate the feasibility of evaluating the nominated indicators.

The aims of this study were to use expert opinion to (i) identify potential indicators of stranded cetacean welfare and survival likelihood, (ii) evaluate those indicators based on their value for assessing stranded cetacean welfare and survival likelihood (i.e. how closely each indicator aligned with welfare state or survival likelihood), and (iii) evaluate how easy/practical each indicator is to measure at cetacean stranding events. Additionally, we aimed to explore expert opinion on the affective experiences that may be inferred if particular animal-based indicators in stranded cetaceans were observed. Development of these indicators will allow for unambiguous assessments of stranded cetaceans, providing measurable objectives for conservation goals which integrate animal welfare.

## 2. Material and methods

This study forms part of a wider research project using expert opinion to characterize and facilitate the improved assessment of stranded cetacean welfare and survival. Detailed methods are described in [17], and key points are summarized here.

This project was evaluated by peer review and assessed as low risk according to criteria set by Massey University Human Ethics Committees (Notification number: 4000023382). All participants provided their informed consent to participate in this study.

### 2.1. Recruitment and characterization of expert participants

Potential participants were identified as experts in the fields of cetacean biology/ecology or wild animal welfare by searching the peer-reviewed literature, documents relating to relevant workshops, and stranding network lists. All participants (*n* = 168) were invited (electronic supplementary material, S1) to complete two questionnaires in 2021, separated by a period of three weeks.

### 2.2. Questionnaire design and implementation

A two-round Delphi process was conducted online using the questionnaire tool Qualtrics [58] to identify potential indicators of stranded cetacean welfare and survival likelihood. Expert opinions on valuable (how closely each indicator aligned with welfare state or survival likelihood) and practical (how feasible each indicator is to measure at strandings) indicators were elicited using an exploratory sequential mixed method [59], with the findings from the first round informing the development of the second round. However, there was no requirement for participants to complete both, and some participants who provided scores in the second round may not have been involved in generating the questions being scored and vice versa.

No identifiable data were collected, ensuring full anonymity. Demographic information was collected to characterize the study population and assess the effect of expertise on the proposed indicators. Experts were asked to self-identify their area of expertise as either: ‘cetacean expert (including cetacean conservation and biology)’, ‘animal welfare expert (including animal welfare science, welfare/animal ethics)’, ‘cetacean expert with knowledge and/or focus on welfare’, ‘animal welfare expert with knowledge and/or focus on cetaceans’, ‘veterinarian’ or ‘other’.

Each questionnaire was split into two sections, each with similar questions: the first section related to the welfare of stranded cetaceans and the second to their survival likelihood. The first questionnaire used mainly unstructured, open-ended questions (electronic supplementary material, S2), allowing participants to provide opinions and elaborate on thoughts regarding indicators of welfare and survival. Experts were asked to identify any measurable and/or observable indicators they felt could be used to assess the welfare state or likelihood of survival of stranded cetaceans. These indicators could be animal-based, such as behaviour, or resource-/management-based, such as weather conditions or human intervention. Participants could provide additional comments if desired. The authors' conceptualization of animal welfare state and likelihood of survival were not provided to ensure that the experts' own understanding of these concepts was captured [17].

The indicators suggested were transcribed intelligent verbatim (i.e. spelling/grammar was corrected) and reviewed independently by the primary author using reflexive thematic analysis to generate common themes from the qualitative data [60,61]. These themes were reviewed by the research team to finalize the major themes for welfare and survival likelihood (electronic supplementary material, S3) that were subsequently used in the development of the second questionnaire (electronic supplementary material, S4). A maximum of 20 major themes per question were presented to participants as ‘categories’ for scoring in the second round, maximizing data collection while minimizing questionnaire fatigue [62]. These categories were identified based on being commonly proposed by participants, as well as being identified as the most relevant and important aspects for the study by the research team [63].

In the second questionnaire, participants were asked to score each of the categories in closed-ended questions with scalar (0–10) responses (S3) [64] based on their value for assessing welfare or survival likelihood, where ‘0 = little/no value’, ‘5 = some value’ and ‘10 = great value’. Participants were also asked to score how easy/practical each indicator category would be to measure at strandings, where ‘0 = difficult to measure’, ‘5 = may be measurable depending on skills/equipment’ and ‘10 = easy to measure’. They could also select the option ‘Don't know’ if they felt they did not have sufficient knowledge to score a particular category. Their thoughts about any barriers to measuring the indicator categories were solicited using an open-ended question.

For categories reflecting animal-based indicators of cetacean welfare, participants were asked to suggest any affective (mental) experience(s) that may be inferred from observation of the indicator. Some of the indicator categories generated through the reflexive thematic analysis of answers from questionnaire 1 comprised multiple individual indicators (electronic supplementary material, S5 table S2). For example, the category ‘Abnormal movements and behaviours’ included *arching*, *thrashing*, *straining*, *agitated movements* and others. For such composite categories, the question of affective experience was asked of each of the individual indicators separately, e.g. what affective experience might be inferred from observation of arching. These were open questions, allowing participants to provide all suggestions they felt were relevant.

### 2.3. Analysis of data from questionnaire 2

The median scores collected were analysed for each category to quantify and rank the indicators, based on their perceived value and practicality for assessing stranded cetacean welfare or survival at stranding events. Higher median scores for categories reflected greater value for assessing, or more easy/practical measurement of, that indicator for welfare or survival. Responses of ‘Don't know’ were not included when calculating median scores.

While quantitative data were collected on continuous scales [65], the responses were pooled into four groupings (score: ‘0–3.99’; ‘4–6.99’; ‘7–10’; ‘Don't know’) to evaluate the level of consensus among participants for each theme. At least 70% of participants had to score a category within the same grouping for consensus to be reached [53,56,66].

Qualitative data reflecting participants' views on barriers to measuring welfare and survival likelihood indicators were investigated using reflective thematic analysis to collate common themes [60,61]. These themes are presented to provide context for the interpretation of experts’ views on the ability to measure the welfare/survival likelihood indicators.

The welfare categories provided were classified based on being welfare status or welfare alerting indicators. These categories were subsequently sorted by the research team into the three physical/functional domains and one situation-related (external conditions) domain of a modified version of the Five Domains Model [26]. Additionally, the qualitative data generated on potential affective experiences were summarized; any term mentioned once for each indicator was included in the summary (electronic supplementary material, S5 table S2), even if not considered an affective experience by the authors. The most common suggestions identified were tallied based on the number of unique mentions for each indicator category (electronic supplementary material, S5 table S1).

Linear discriminant analysis (LDA) was conducted using package MASS [67] in R (v. 1.2.5033) to visualize the effect of participants' self-identified expertise on the scores for value and ease/practicality of measuring welfare and survival likelihood indicator categories. When an expert responded ‘Don't know’ for a particular category, data imputation was applied by calculating the average score of the expertise group across that category. Orthogonal axes were generated to maximally separate the six expertise groups based on participant scores. Visual representations of the differences and similarities among expertise groups were provided using the first two axes of each LDA, prepared using the package ggplot2 [68].

## 3. Results

International experts were invited to participate (*n* = 168) in both questionnaires; the first and second questionnaires had a response rate of 40.5% (*n* = 68) and 37.5% (*n* = 63), respectively. Variation among expertise and regional representation was minimal between surveys (table 1; [17]).

**Table 1.** Number of expert participants in each self-defined expertise category and their current region of work across two questionnaires.

expertise	questionnaire 1	questionnaire 2	region	questionnaire 1	questionnaire 2
cetacean conservation and biology	26% (*n* = 18)	25% (*n* = 16)	Europe	40% (*n* = 27)	41% (*n* = 26)
veterinary medicine	24% (*n* = 16)	32% (*n* = 20)	Oceania	25% (*n* = 17)	30% (*n* = 19)
animal welfare science/ethics	16% (*n* = 11)	14% (*n* = 9)	North America	22% (*n* = 15)	16% (*n* = 10)
cetacean biology with a focus on welfare	16% (*n* = 11)	19% (*n* = 12)	South America	6% (*n* = 4)	6% (*n* = 4)
animal welfare with a focus on cetaceans	3% (*n* = 2)	5% (*n* = 3)	Asia	4% (*n* = 3)	2% (*n* = 1)
other: strandings response/broader ecology	15% (*n* = 10)	5% (*n* = 3)	Central America	1% (*n* = 1)	3% (*n* = 2)
			Africa	1% (*n* = 1)	2% (*n* = 1)

### 3.1. Developing indicators of stranded cetacean welfare

#### 3.1.1. Perceived value of welfare indicators

**Figure 1.**
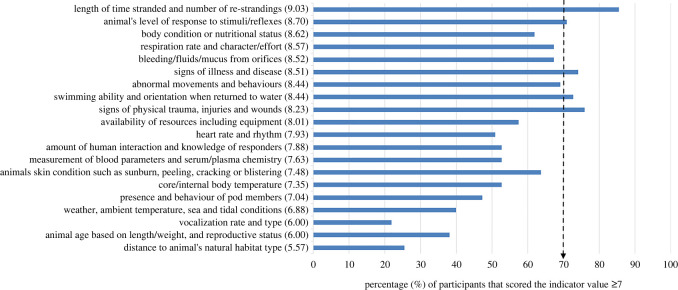
The perceived value of the 20 welfare indicator categories generated from reflexive thematic analysis of expert suggestions, ranked in order of the median score (in brackets) on the *y*-axis, with percentage of experts scoring the theme greater than or equal to 7/10 on the *x*-axis. The dashed arrow represents the consensus level i.e. 70% of experts (*n* = 44/63) scored the indicator as greatly valuable. The category labels have been simplified to fit on the figure, see electronic supplementary material, S3 table S1 for full labels.

Forty-nine themes reflecting indicators for assessing cetacean welfare were generated from questionnaire 1 responses (electronic supplementary material, S3 table S1). Twenty of these themes were provided in the second round for scoring as categories. Sixteen of the 20 indicator categories presented in questionnaire 2 were perceived to have ‘great value’ (median scores ≥7; figure 1). Five categories achieved consensus as being of great value (scores ≥7) by at least 70% (*n* = 44/63) of the experts, these included one resource-based indicator (‘length of time stranded and number of re-strandings'), and four animal-based indicators (‘signs of physical trauma, injuries and wounds’; ‘signs of illness and disease’; ‘swimming ability and orientation when returned to water’; ‘animal's level of response to stimuli/reflex’). The indicator perceived as most valuable for assessing welfare state, based on the percentage of experts scoring it greater than or equal to 7, was ‘length of time stranded and number of re-strandings’ while the least valued indicator of the 20 presented was ‘vocalization rate and type’.

#### 3.1.2. Perceived practicality of welfare indicators

**Figure 2.**
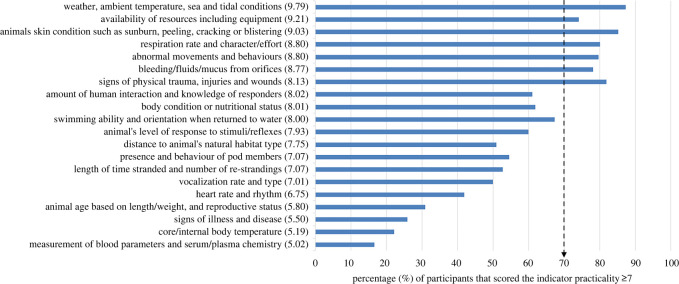
The perceived practicality of the 20 welfare indicator categories generated from reflexive thematic analysis of expert suggestions, ranked in order of the median score (in brackets) on the *y*-axis, with percentage of experts scoring the theme greater than or equal to 7/10 on the *x*-axis. The dashed arrow represents the consensus level i.e. 70% of experts (*n* = 44/63) scored the indicator as practical to measure. The category labels have been simplified to fit on the figure, see electronic supplementary material, S3 table S1 for full labels.

Fifteen welfare indicator categories had a median score of greater than or equal to 7, suggesting that experts viewed them as easy/practical to measure at stranding events (figure 2). Seven categories reached expert consensus as being easy/practical to measure (score ≥7; figure 2). These included two resource-based indicators (‘weather, ambient temperature, sea and tidal conditions’ and ‘availability of resources including equipment’) and five animal-based indicators (‘bleeding/fluids/mucus from orifices’; ‘abnormal movements and behaviours including arching, thrashing, straining, trying to move, agitated movements, slapping flukes, tremors/shivering’; ‘respiration rate and character/effort’; ‘signs of physical trauma, injuries and wounds’; and ‘animal's skin condition such as sunburn, peeling, cracking or blistering’). The indicator considered easiest to measure, based on the percentage of experts scoring it as greater than or equal to 7, was ‘weather, ambient temperature, sea and tidal conditions’ while ‘measurement of blood parameters and serum/plasma chemistry’ was deemed the most difficult.

Notably, of the most valuable welfare indicator categories (scores ≥7 by at least 70% of the experts (*n* = 44/63)), only one reached consensus as being easy/practical to measure: ‘signs of physical trauma, injuries and wounds’. However, of the other easily measurable welfare indicators (i.e. reaching consensus), all except one (‘weather, ambient temperature, sea and tidal conditions’) were considered to also be valuable (≥7) by over 50% (*n* = 32/63) of the experts.

#### 3.1.3. Barriers to measuring indicators of stranded cetacean welfare

Twenty-eight of the 63 participants (44%) in questionnaire 2 identified major barriers to measuring indicators of cetacean welfare state. From reflexive thematic analysis of qualitative responses, three major themes were interpreted:

— skills/training/knowledge of responders,
— lack of data and ability to interpret parameters and assess changes from baseline, and
— difficulties in assessing each indicator due to equipment/situation at stranding event.

The main barrier regarding the measurement of welfare indicators highlighted by 20 experts (71%) who provided a response to the question was the availability of well-trained, experienced and knowledgeable personnel. For example, one participant noted:

Having […] properly trained users to make accurate measurements of certain things (vocalisations, heart rate, taking blood samples) and ability to obtain results during the stranding event to have any meaningful use for decision making.

It was also emphasized that for many parameters, both physiological and behavioural, baseline data against which to compare measures taken during a stranding are lacking, which limits the ability to identify welfare-relevant deviations. One participant noted:

There is still a lot we do not know about cetaceans (e.g.: normal heart rates and blood parameters of different species in the wild) to form baseline ‘normal’ data for them that samples/data from stranded cetaceans can be compared to…

Additionally, it was mentioned that the specialized equipment required and features of the stranding event (e.g. number of animals, size of animals, position/location when stranded) would affect which indicators could be measured *in situ*. One participant noted:

Rectal temperature may not be safe or possible […]. Heart rate and rhythm easy enough on small cetaceans but requires ECG for large cetaceans. Point of care blood analysers may be used in field situations but some field situations are not conducive even for those instruments.

#### 3.1.4. Classification of welfare indicators using the Five Domains Model

The 20 indicator categories generated from reflexive thematic analysis of questionnaire 1 data, included 13 welfare status indicators and seven welfare alerting indicators. These categories included representative indicators fitting into all three of the physical/functional domains and one situation-related domain in the Five Domains Model framework (table 2). Four of the five indicators that reached expert consensus as being valuable were welfare status indicators, with three in the health domain and one in the behavioural interactions domain (table 2). Five of the seven indicators that reached consensus as being practical to measure were welfare status indicators, with one in the physical environment domain, three in the health domain and one in the behavioural interactions domain (table 2).

**Table 2.** The 20 welfare status and welfare alerting indicator categories generated from reflexive thematic analysis of expert suggestions, categorized according to the Five Domains Model [26].

domain	indicator types	
	welfare status	welfare alerting
1: nutrition	body condition or nutritional status	animal age based on length/weight or reproductive status
2: physical environment	animal's skin condition such as sunburn, peeling, cracking or blistering, desiccation^†^ core/internal body temperature	length of time stranded and number of re-strandings* availability of resources including equipment^†^ weather, ambient temperature, sea and tidal conditions^†^ distance to animal's natural habitat type
3: health	signs of physical trauma, injuries and wounds*^,†^ signs of illness and disease* respiration rate and character/effort^†^ bleeding/fluids/mucus from orifices^†^ measurement of blood parameters and serum/plasma chemistry heart rate and rhythm or function animal's level of response to stimuli/reflexes as a reflection of its level of awareness, alertness or consciousness*	
4: behavioural interactions	swimming ability and orientation when returned to water* abnormal movements and behaviours including arching, thrashing, straining, trying to move, agitated movements, slapping flukes, tremors/shivering^†^ vocalization rate and type	presence and behaviour of pod members amount of human interaction and knowledge of responders

Symbols indicate categories that reached expert consensus for value* and practicality^†^.

#### 3.1.5. Inferring affective experience from welfare status indicators

There were 26 individual indicators extracted from the 13 welfare status categories (electronic supplementary material, S5 table S2). Forty-three (68%) of the total 63 participants provided a response to at least one indicator for the question on affective experiences (median: 41, range: 33–43).

Most of the inferred affective experiences were negatively valenced, i.e. unpleasant experiences (electronic supplementary material, S5 table S1). The most suggested affective experiences, based on the number of indicators that they were mentioned for, were ‘stress’, ‘distress’, ‘pain’ and ‘fear’, with at least one of these suggested for every indicator. Additionally, ‘dizziness’ and ‘lethargy’ were suggested for several indicators, while other affective experiences were indicator specific, such as ‘breathlessness’ which was mentioned only in relation to respiratory indicators (electronic supplementary material, S5 table S2). Furthermore, some of the affective experiences mentioned for ‘reduced respiration rate’, ‘abnormal swimming movements’, ‘fluke slapping’ and ‘vocalization’ could be considered positively valenced, including ‘relaxed’, ‘resting’, ‘calm’, ‘excitement’ ‘happy’ and ‘safety’ (electronic supplementary material, S5 table S2). The most commonly suggested affective experiences for each indicator are reported in electronic supplementary material, S5 table S1 (see electronic supplementary material, S5 table S2 for all suggestions provided by experts).

Instead of providing an affective experience, experts sometimes suggested potential underlying causes of the indicator being displayed; for example, for physiological indicators ‘agonal’, ‘adrenaline’, ‘pruritis’, ‘decompensating’, ‘hyperthermia’; for behavioural indicators ‘effort to escape’ and ‘avoidance behaviour’ (see electronic supplementary material, S5 tables S1 and S2). These responses have been collated under the corresponding physical/functional (Domains 1 to 3) or situational (Domain 4) domain (electronic supplementary material, S5 table S1) to distinguish them from commonly accepted affective experiences (Domain 5: Mental state). Additionally, two veterinarians indicated that they were not comfortable with providing a potential affective experience that may be inferred when observing a particular welfare status indicator. For example, one veterinarian stated:

Personally I feel like great care must be taken when imposing our assumptions on the experience of an animal – I am quite reluctant to fill in any of the above as I do not believe we have the right to comment on many of those as to how the animal experiences the situation. I would even be reluctant to comment on how another human would experience those conditions as there is so much individual variability.

### 3.2. Developing indicators of likelihood of stranded cetacean survival

#### 3.2.1. Perceived value of survival likelihood indicators

**Figure 3.**
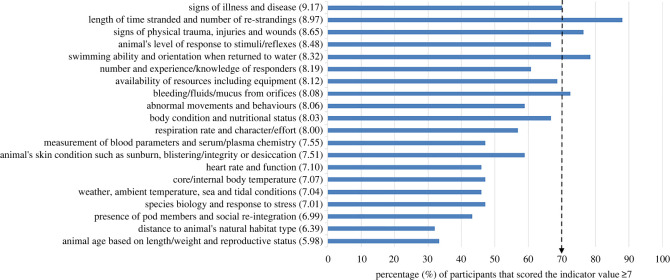
The perceived value of the 20 survival likelihood indicator categories generated from reflexive thematic analysis of expert suggestions, ranked in order of the median score (in brackets) on the y-axis, with percentage of experts scoring the theme greater than or equal to 7/10 on the *x*-axis. The dashed arrow represents the consensus level i.e. 70% (*n* = 44/63) of experts scored the indicator as highly valuable. The category labels have been simplified to fit on the figure, see electronic supplementary material, S3 table S2 for full labels.

Forty themes on assessing likelihood of survival were generated from reflexive thematic analysis of participant responses in questionnaire 1 (electronic supplementary material, S3 table S2). Twenty of the major themes were provided for scoring as categories in the second round. Seventeen of the 20 indicator categories presented in questionnaire 2 were perceived to have ‘great value’ (median scores ≥7; figure 3) for assessing survival likelihood.

Five indicator categories achieved consensus as being of great value (scores ≥7) by at least 70% (*n* = 44/63) of the experts, including one resource-based indicator (‘length of time stranded and number of re-strandings’) and four animal-based indicators (‘signs of physical trauma, injuries and wounds’; ‘signs of illness and disease’; ‘swimming ability and orientation when returned to water’; ‘bleeding/fluids/mucus from orifices’, figure 3). The most valued indicator category for survival likelihood, based on the percentage of experts scoring it greater than or equal to 7, was ‘length of time stranded and number of re-strandings’ while the least valued indicator category was ‘distance to animal's natural habitat type’ (figure 3).

#### 3.2.2. Perceived practicality of survival likelihood indicators

**Figure 4.**
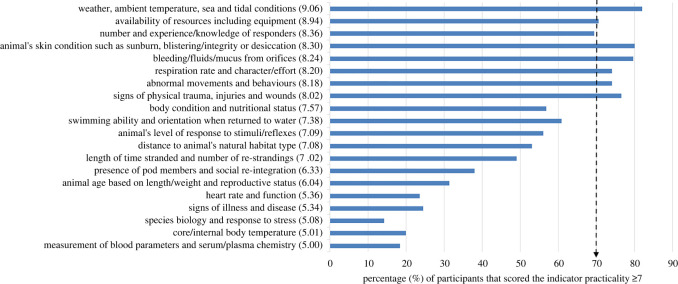
The perceived practicality of the 20 survival likelihood indicator categories generated from reflexive thematic analysis of expert suggestions, ranked in order of the median score (in brackets) on the *y*-axis, with percentage of experts scoring the theme greater than or equal to 7/10 on the *x*-axis. The dashed arrow represents the consensus level i.e. 70% (*n* = 44/63) of experts scored the indicator as practical to measure. The category labels have been simplified to fit on the figure, see electronic supplementary material, S3 table S2 for full labels.

Thirteen survival likelihood indicator categories were perceived to be easy/practical to measure at stranding events (median score ≥7; figure 4). Seven indicator categories reached expert consensus (≥70%; *n* = 44/63) as being easy/practical to measure. These included two resource-/management-based indicators (‘weather, ambient temperature, sea and tidal conditions’ and ‘availability of resources including equipment’) and five animal-based indicators (‘bleeding/fluids/mucus from orifices’; ‘abnormal movements and behaviours including arching, thrashing, straining, trying to move, agitated movements, slapping flukes, tremors/shivering’; ‘respiration rate and character/effort’; ‘signs of physical trauma, injuries and wounds’; and ‘animal's skin condition such as sunburn, peeling, cracking or blistering’). The category considered most practical for measurement, being scored greater than or equal to 7 by the highest percentage of experts, was resource-based (‘weather, ambient temperature, sea and tidal conditions’) but this indicator was only considered highly valuable by 46% (*n* = 29/63) of experts. The least practical to measure, based on the percentage of experts scoring greater than or equal to 7, was ‘species biology and response to stress’.

Two of the most valuable indicator categories reached consensus as being easy/practical to measure (‘signs of physical trauma, injuries and wounds’ and ‘bleeding/fluids/mucus from orifices’). However, of the other easy/practical-to-measure indicator categories (i.e. reaching consensus), all except one (‘weather, ambient temperature, sea and tidal conditions’) were considered by the experts to be greatly valuable (≥7) indicators of survival likelihood by over 50% (*n* = 32/63) of the experts.

#### 3.2.3. Barriers to measuring survival likelihood indicators

Few participants (19%, *n* = 12/63) contributed major barriers to measuring the indicators of survival likelihood. Three major themes were interpreted from reflexive thematic analysis of these qualitative responses:

— skills/training/knowledge of responders,
— lack of data and ability to interpret parameters and assess changes from baseline, and
— difficulties in assessing each indicator due to equipment/situation at stranding event.

The main barrier regarding the measurement of survival likelihood indicators highlighted by six experts (50%) who provided a response to the question, was the availability of well-trained, experienced and knowledgeable personnel. For example, one participant noted:

[…] External signs should be easier to record and assess, especially when a remote expert can see photographs taken at the stranding – a barrier here is ensuring that the photographer takes photos that are useful and not just for social media use. Having the right people looking for indicators is imperative – they need to be trained and informed about cetaceans.

It was also noted that baseline data against which to compare measurements taken during a stranding are lacking. One participant noted:

[…] having access to ‘normal’ baseline data for different species in the wild to compare with (e.g.: blood parameters, heart rates etc).

The need for specialized equipment and complexities of undertaking measurements at stranding events were also highlighted by several participants:

Any internal indicators can only really be taken with the right equipment – core temperature for example. Vets may carry thermometers but probably not a thermistor probe long enough to measure core temperature. […]

### 3.3. Potentially valuable and practical indicators of welfare and survival likelihood for future assessments

Thirty-seven themes (75.5%) generated from reflexive thematic analysis of questionnaire 1 responses were consistent for both welfare and survival likelihood (electronic supplementary material, S3). In addition, 18 of the 20 indicator categories (90%) presented in questionnaire 2 for scoring were similar for welfare and survival likelihood. The indicator category that applied only to welfare was ‘vocalization rate and type’, while the indicator category that applied only to survival was ‘species biology and response to stress’.

Four of the five (80%) indicator categories that reached consensus based on their perceived value were the same for welfare and survival likelihood (‘length of time stranded and number of re-strandings’, ‘signs of physical trauma, injuries and wounds’, ‘signs of illness and disease’ and ‘swimming ability and orientation when returned to water'). Additionally, all seven indicator categories that reached consensus based on being easy/practical to measure were the same for both welfare and survival likelihood.

The indicator categories which were perceived to be greatly valuable (median scores ≥7) and easy/practical to observe and/or measure (median scores ≥7) for both welfare and survival are collated in table 3.

**Table 3.** Indicator categories perceived by experts (*n* = 63) as valuable (median score ≥7) and measurable (median score ≥7) for both stranded cetacean welfare and survival likelihood. Type of indicator is provided to highlight those potentially providing direct welfare status information. For each, some basic recommendations for observation and/or measurement of the indicator using video and/or photographs are provided based on the authors experience at strandings (Massey University 2021, unpublished data) and previous research on cetacean welfare [69] or strandings [13].

indicator	type of indicator	method of observation/measurement	
abnormal movements and behaviours including arching, thrashing, straining, trying to move, agitated movements, slapping flukes, tremors/shivering	status	video	continuously film cranio-laterally with entire animal body in frame if possible. Note what behaviours are occurring, how many times they occur (frequency) and for how long (duration). Note if other events e.g. human intervention, are occurring around animal simultaneously
animal's level of response to stimuli/reflexes as a reflection of its level of awareness, alertness or consciousness	status	video	when animal's eye open walk around, along length of animal to see if eye follows movement. If instructed by experts, gently touch around blowhole to see if blowhole tightens closed, gently touch at edge of corner of eye to see if there is a blink response
animals skin condition such as sunburn, peeling, cracking or blistering	status	photograph/video	estimate the percentage of the body that has skin blistering and/or peeling, estimate depth of blisters based on layers of skin involved e.g. superficial sloughing
bleeding/fluids/mucus from orifices	status	video	regular observation of all accessible orifices (e.g. mouth, blowhole, eyes) and note signs of blood, mucus or other fluids including appearance of colour and consistency
body condition or nutritional status	status	photograph/video	take photographs cranio-ventrally at head level towards flukes, observe body shape in epaxial section and thoracic wall e.g. for protrusion of ribs. Photograph cranio-laterally at head level to observe any concavity in the nuchal crest (area dorsal to blowhole)
respiration rate and character/effort	status	video	count the number of times the animal breathes during a 5 min period, observe whether the animal breathes out and then in, are they any noises when the animal breathes, does the animal make effort e.g. move each time it breathes?
signs of physical trauma, injuries and wounds	status	photograph/video	look for any injuries, make note of how many an animal has, if they are bleeding: superficial or deep wounds, observe for any awkward looking positions e.g. pectoral fins that are up and out instead of close to body and down, curved peduncle position that does not change
swimming ability and orientation when returned to water	status	video	after a period of acclimation in water is the animal able to remain upright alone, does it roll more to one side? If it is swimming, does it swim straight or in circles?
amount of human interaction, number and knowledge of responders	alerting	video	count number of humans, what interactions are they having with the animal, what training they have
availability of resources including equipment	alerting	photograph/video	what equipment is being used e.g. sheets, buckets, refloating pontoons? Also discuss with local authorities, strandings network about available equipment
length of time stranded and number of re-strandings	alerting	video	gather information from field responders about when animal was first found stranded, how many times has it been refloated? Film any refloating procedures and subsequent behaviour including re-strandings

### 3.4. Effect of participant expertise on perceived value and practicality of indicators

The LDAs revealed overlap of expertise groups in the scoring of welfare and survival likelihood indicator categories in terms of both value and practicality. This suggests that self-reported expertise did not have a major effect on the scoring of indicator categories. Specific results from the LDAs are presented in the electronic supplementary material, S6.

## 4. Discussion

As cetacean stranding events are predicted to increase globally, it is critical that management decisions be informed by both the likelihood that the animal will survive post-refloating and the impacts that both the stranding and human intervention have on the animals' welfare state (overall affective experience). However, empirical data to inform such assessments is currently lacking [45,47]. This study has provided face validity (alignment between indicators and welfare state/survival likelihood) to the first comprehensive list of indicators perceived by international, interdisciplinary experts, to be valuable and practical to measure, for assessments of stranded cetacean welfare and survival likelihood. These indicators should now be further evaluated at cetacean stranding events to confirm their feasibility and their predictive value for survival likelihood. Furthermore, this study provides potential affective states that cetaceans may experience during a stranding event, based on expert opinion. Given the paucity of empirical data available globally to assess stranded cetacean welfare and survival likelihood, this represents an important step in the development of a systematic assessment framework to support holistic, scientifically informed decision-making.

Notably, the indicator categories arising from expert opinions highlight the inextricable link between welfare and survival likelihood for stranded cetaceans, with many of the same indicators suggested for both concepts. This emphasizes that improvements in knowledge and ability to assess such indicators will advance evaluation of both welfare and survival likelihood of stranded cetaceans. However, the variable affective experience suggestions provided by experts for welfare status indicators highlight the importance of investigating how indicators correlate among differing variables [70]. For example, further work correlating behavioural expression with factors such as physical state, time stranded, types of intervention and actual survivorship should be considered research priorities.

### 4.1. Using expert opinion to develop valuable and measurable indicators of stranded cetacean welfare and survival likelihood

Indicators that emerged as both valuable (how closely each indicator aligned with welfare state or survival likelihood) and practical (how feasible the indicator is to measure) for assessments, based on the median scores and achieving consensus, were similar for welfare and survival. It is useful that these experts report that the same indicators can be used to assess both welfare state and likelihood of survival since this will allow for the development of an assessment framework that considers both concepts concurrently. Importantly, many of the indicators relate to the concepts and concerns for stranded cetacean welfare and survival likelihood that were highlighted by the same group of experts [17]. Therefore, application of these indicators would ensure that both immediate welfare impacts and survival likelihood of stranded cetaceans scientifically inform decision-making regarding reflotation, palliative care, or humane killing i.e. euthanasia [5,17].

The most valuable indicators for welfare which reached consensus, included four welfare status indicators: ‘physical trauma, injuries and wounds’, ‘animal's level of responsiveness’ and ‘illness/disease’ in the health domain (D3), and ‘swimming ability’ in the behavioural interactions domain (D4). The fifth indicator that reached consensus was welfare-alerting and resource-/management-based: ‘length of time stranded’ in the physical environment domain (D2). Four of these indicators were also considered to be of great value and reached consensus for survival likelihood. The indicator that did not reach consensus for survival likelihood was ‘animal's level of responsiveness’, though this was still considered to be of great value (median score: 8.5). Additionally, for survival likelihood another animal-based indicator ‘bleeding/fluids/mucus from orifices’ was of great value and reached consensus. Notably, this indicator was also considered to be greatly valuable for welfare (median score: 8.5), being a welfare status indicator in the health domain (D3).

Of the welfare indicators that reached consensus as being greatly valuable, only one was considered to also be practical to measure based on consensus ('physical trauma, injuries, wounds'). Likewise, for the valuable survival likelihood indicators that reached consensus, only two were considered to be practical to measure based on consensus: ‘physical trauma, injuries, wounds’ and ‘bleeding/fluids/mucus from orifices’. However, when considering the median scores of the other valuable indicators, all but one ('illness/disease') were considered practical to measure (median score ≥7). This suggests that most of the valuable indicators would be assessable at stranding events.

Those indicators that were considered practical to measure based on consensus were the same for both welfare and survival likelihood. These included five animal-based indicators (‘animal's skin condition’, ‘abnormal behaviour’, ‘respiration rate’, ‘bleeding/fluid/mucus from orifices’ and ‘physical trauma, injuries, wounds’) and two resource-/management-based indicators ('weather’ and ‘availability of resources'). Notably, all these practical indicators, except ‘weather’, were also considered greatly valuable based on their median scores (≥7). In this case, weather was probably not considered to be valuable since it is not directly related to welfare state or survival likelihood. However, it may still provide additional risk-related information, since concerns likely to have negative effects on stranded cetacean welfare and survival likelihood included hyperthermia and sunburn [17], which are more likely to occur on hot and/or sunny days [38].

Two indicators did not reach consensus for value or practical measurement but based on median scores (≥7) were still considered by experts as both greatly valuable and practical to measure for both welfare and survival likelihood. These were the animal-based indicator ‘animal body condition’, which would represent welfare status in the domain nutrition (D1), and the resource-/management-based indicator ‘human interaction, number and knowledge of responders’, which would be considered as welfare alerting in the domain behavioural interactions (D4).

Despite only a third of experts reporting animal welfare knowledge, the complement of animal-based indicators recommended by the experts facilitates holistic assessment of animal welfare state and survival likelihood. Consistent application of the indicators considered to be both valuable and practical would ensure scientifically informed decision-making at stranding events. The fact that the representative indicators suggested by experts fit in all four domains of the Five Domains Model [26], emphasizes its potential in the development of a structured assessment framework specific to stranded cetaceans. We tentatively suggest that the indicator categories provided in table 3, may be potential candidates for assessment of both welfare and survival likelihood at stranding events, based on being considered valuable and practical by experts (median scores ≥7). Additionally, these indicators are probably assessable via video and could be used by remote experts in an evaluation (table 3); a factor emphasized to be important by experts in this study since there are often limited trained/knowledgeable personnel at stranding events.

Those indicators relating to an animal's physical state (i.e. aligned to Domains 1 to 3) could provide information about why an animal may have stranded and how it is likely to cope with prolonged physiological stress. Poor body condition, physical trauma/injury and bleeding/fluid from orifices are probably linked to negatively valenced welfare states [45,69,71] and can affect the outcome of strandings due to the detrimental effect on survival [38,72,73]. Objective assessment of these indicators may be limited where there are incomplete data available, such as for visual assessment of body condition [74], and difficulties in diagnosing internal injuries [75], which experts have highlighted as potential barriers to assessments. Despite these difficulties, some of these suggested indicators may correlate with certain management decisions. For example, haemorrhaging from orifices is considered an indication of significant internal injury, and a decision to euthanize is often implied due to the likely compromised welfare and low survival likelihood of the animal [5,76,77].

Indicators were also generated that provide suggestion of how being out of the water is probably affecting an animal in terms of its skin condition, respiratory rate and character/effort. Assessment of skin condition could provide information on the animals physiological state, and skin blistering is suggested for use in the decision-making process for reflotation versus euthanasia due to the likely impact upon both welfare and survival likelihood [5,78,79]. Respiration rate and character will vary depending on the state of the animal; high respiratory rates (greater than 6 breaths min^−1^) in delphinids are suggestive of stress [42,80]. Other respiratory abnormalities, including a delay of a few seconds between expiration and inspiration, and prolonged apnoea are considered indicative of shock [42]. Respiratory impairment is survival-critical, thus impacting upon an animals ability to survive, but is also likely to induce the negatively valenced welfare state of breathlessness, aligned to a health condition in a survival-related domain (D3) [81,82].

Behavioural indicators probably reflect how the animal's ability to exercise agency or choice is impacted by its unnatural situation and can be used to infer how it is experiencing its current situation. Unfortunately, there has been only one ethological study undertaken on stranded cetaceans [83], therefore assessing abnormal movements and what they may reflect in these animals is currently limited. Nevertheless, some behaviours have been noted previously as informing refloat decisions based on animal disposition [15,16,84]. For example, arching is assumed to be a sign of significant physiological stress [85] and requires animals undergoing health assessments to be released immediately if observed, due to the negative impact on welfare and potential detrimental effect on survival [85,86]. Another indicator that was generated and used in capture-release health assessments is animal responsiveness. In these assessments, animals are required to be alert and responsive before human intervention can be undertaken [85]. Animals that begin alert but have progressively reduced reflexes and dull eyes are likely to be deteriorating [42,85,87], and stranded animals displaying a loss of reflexes should be considered candidates for euthanasia due to their welfare compromise and low survival likelihood [5,76]. Finally, swimming ability was also suggested; however, this can only be assessed once an animal is refloated. Therefore, it should be considered as part of a final assessment to ensure that a refloated animal is ready to be released, rather than as an indicator of animal viability [39].

Experts emphasized that there is limited baseline information for stranded cetaceans against which to compare physiological or behavioural deviations and identify relevant welfare and/or survival compromise. Therefore, further data collection and correlation among indicators and additional strandings context will be required to ensure rigorous evaluation [70]. Experts also highlighted the importance of trained personnel to accurately interpret behavioural and physiological parameters and evaluate these in the context of each stranding situation; unfortunately, this is currently limited by the variable availability of knowledgeable, trained personnel at many stranding events [17,39].

Three resource-/management-based indicators considered valuable and practical to assess both welfare and survival likelihood included the length of time stranded/number of re-strandings, and features related to human intervention in terms of knowledge and available resources, such as equipment. These indicators provide relevant information on the potential risks of each stranding situation to animal welfare and survival likelihood [12,88]. For example, the length of time stranded and number of re-strandings will affect the level of compounding damage that a stranded cetacean undergoes since prolonged and/or multiple strandings probably cause sustained physiological stress responses [72,89]. Therefore, longer, or numerous strandings are expected to increase the duration and intensity of negative welfare states and detrimentally impact survival likelihood.

Inappropriate human intervention related to responder lack of knowledge and/or unsuitable equipment can lead to additional animal injury and/or increased mortality [43], adversely affecting both welfare and survival likelihood. By contrast, appropriate intervention with suitable equipment may minimize harm and improve survival likelihood by reducing the amount of time stranded [72]. However, the impact upon animal welfare is more complex to understand, since cetaceans, as wild animals unaccustomed to close human contact, may experience any form of human interaction as a threatening experience, negatively impacting welfare [26]. Nevertheless, humans may also reduce the potential for significant welfare compromise caused by stranding-associated factors, such as hyperthermia or sunburn, by providing shade and cooling water [38].

We recommend that future studies prioritize data collection at stranding events to identify observable and/or measurable indicators, evaluate their feasibility for use in assessments and validate their functional impact to inform intervention decisions [90]. Such systematic data collection would also provide a baseline against which to examine for abnormalities. Improved data collection would enable an understanding of which indicators could be assessed by experts remotely via video recordings and/or photographs. Once such investigations are undertaken, extended work with experts should assess the reliability and validity of each indicator [54,55] for assessing stranded cetaceans.

### 4.2. Inferring affective experience from animal-based welfare indicators

This study also provides an initial understanding of potential affective states that experts postulated stranded cetaceans may experience based on impacts in each physical/functional and situation-related domain. These would be included in the fifth domain of the Five Domains Model [26]. Generally, consensus among experts about the valence of affective states inferred by the indicators was evident, with almost all indicators being interpreted as reflecting negative experiences such as ‘stress’, ‘distress’, ‘pain’ and ‘fear’. This is likely to be due to the fact that strandings are understood to be atypical situations where physical disruptions and physiological instabilities can occur [72,89,91] which will probably lead to negative mental/affective states. Additionally, it is likely that experts explicitly linked the welfare indicators to survival and end-of-life decision-making which typically focuses on alleviating suffering.

However, several terms with positive connotations (‘relaxed’, ‘resting’, ‘calm’, ‘excitement’, ‘happy’ and ‘safety’) were offered by experts for the indicators ‘reduced respiration rate’, ‘abnormal swimming movements’, ‘fluke slapping’ and ‘vocalisation’, respectively. Furthermore, both negatively and positively valenced affects were suggested by experts for the indicator vocalization. This could have important implications for decision-making if a vocalization is considered an indication of ‘happy’ or ‘excitement’ due to human interaction rather than a distress call. Notably, for other behavioural indicators there was no clear expert consensus in terms of specific affects, other than generally being negative, probably due to the context of stranding. Increased data collection on a suite of behavioural and physiological indicators should be undertaken at strandings, to allow for correlations to be investigated with additional stranding-related factors such as physical state, time stranded and actual survival. This will ensure valid evaluation of such indicators and appropriate inferences to inform strandings management [70,90].

The same group of experts emphasized an integrated approach for characterizing welfare and evaluating key welfare concerns for stranded cetaceans, including biological function, health, behaviour and affective state [17]. Despite this, some experts did not provide affective experiences, and instead gave descriptions of what an indicator may mean, such as ‘Animal may be slipping away’, or related to a possible medical explanation, such as ‘neurological disease or condition’ (see electronic supplementary material, S5 tables S1 and S2). This suggests that although experts understand animal welfare to be a property of the individual cetacean [17], some experts are not comfortable inferring a subjective mental experience that the cetacean may be enduring. This was explicitly noted by two veterinarians and is a legitimate concern, as there is much scientific research required to provide support for such inferences [27,29].

Importantly, the variation in understanding of what indicators reflect could influence the evaluation of animal welfare at stranding events, particularly in relation to the level of importance given to different outcomes [1]. For example, assessments that focus solely on evaluations of biological function without cautiously interpreting the results in terms of affective state may suggest that animals would survive and result in reflotation, but this does not mean these animals are in a good welfare state or have a life worth living [92]. Crucially, poor welfare state can have conservation implications by impacting fitness parameters [93,94] and long-term survival [95]. According to contemporary concepts of animal welfare, including those generated by this group of experts [17], ‘fitness’ should be understood in terms of how it relates to impacts upon the animal's affective state. Therefore, the potential affective experiences of cetaceans must be considered from objective assessment of a range of indicators, to ensure that holistic approaches to welfare and survival are integrated in the decision-making process [1,2] considering both during and post stranding.

### 4.3. Study considerations

Experts in this study were also asked to characterize the welfare of stranded cetaceans [17], this characterization can be used to infer the validity of the welfare indicators presented here. The same expert group conceptualized stranded cetacean welfare according to contemporary animal welfare science [17], which encompasses physical, behavioural and situation-related factors and how these impact upon affective state. Therefore, it is unsurprising that many welfare and survival indicators aligned. However, if the construct of welfare was understood by participants from differing animal welfare orientations, then these conceptions could have limited the scope of indicators to be considered in an assessment and may have favoured others; this might limit the ability to adequately address welfare concerns and may conflict the conclusions of an assessment [96]. For example, those emphasizing the biological function orientation are more likely to focus on issues affecting health/function (e.g. respiratory disease), but may discount other indicators, such as separation from conspecifics, which do not immediately impact health/function. For social species of stranded cetaceans, this could lead to healthy animals being refloated alone leading to unnatural social isolation which may impact future survival [97]. By contrast, those whose conceptualization of welfare aligns with the natural living concept are more likely to focus on the animal's ability to perform their natural behaviours in terms of their needs and motivation. This is particularly relevant in captive or managed environments, e.g. zoos [98], but in the case of stranded cetaceans is limited in its utility, since the animals are in an abnormal environment unable to perform natural behaviours. Therefore, it is important to understand how welfare has been conceptualized before undertaking an assessment to ensure that the interpretation of outcomes is valid and reliable for the construct being measured, i.e. to ensure that face validity is effectively characterized [96]. Face validity of the welfare indicators presented here can be inferred from the conceptualization of stranded cetacean welfare, which was characterized by the same group of experts [17].

By generating categories through reflexive thematic analysis using experts' verbatim wording, conclusions were able to be drawn from the data rather than approaching it with preconceived ideas [60,63]. However, we acknowledge that the primary researcher (RMB) in particular, had a role in co-generating the categories presented for scoring, and therefore the data created cannot be considered ‘objective’ [99,100]. The primary researcher is a marine biologist focused on cetacean strandings, and therefore has personal experiences and opinions relating to the categories explored. Throughout the questionnaire, experts were provided with opportunities to comment and none were received, suggesting that the experts agreed with the categories presented for scoring, providing some ‘ground-truthing’ to the co-generated data [101].

Participants that were involved in the generation of themes in questionnaire 1, did not have to complete questionnaire 2, and vice versa. This may mean that some experts that responded only to questionnaire 2 may have suggested additional indicators that were not presented for scoring. However, throughout questionnaire 2 experts were able to provide comments, and none were received to suggest experts felt any important categories were missing.

Participants of variable expertise (cetacean biologists, animal welfare scientists and veterinarians) responded to this study, enabling both welfare and conservation focused indicators to be generated. This is important as heterogeneity is understood to lead to improved results when applying group decision-making [102]. Notably, the overlap in the LDAs indicated no major differences existed among expertise in terms of indicator value or practicality scoring (electronic supplementary material, S6). This suggests that, overall, there was consensus among expertise on those indicators that should be further investigated as part of the development of a framework to assess stranded cetacean welfare and survival likelihood.

Effort was made to ensure that experts were identified globally and provide robust regional representation. Nevertheless, fewer experts were identified in South and Central America, Asia and Africa. This bias is also reflected in stranding response networks globally [103] and is due in part to socio-economic factors that may decrease the number of publications, workshops and responders in these regions [104,105].

## 5. Conclusion

This study generated a range of animal-based and resource-/management-based indicators specific to stranded cetaceans that were considered valuable and practical to measure. The complement of indicators generated and ranked by the interdisciplinary, international expert group reflect a holistic approach to assessing both welfare and survival likelihood. Importantly, the generated indicators emphasized the inextricable link between welfare and survival likelihood for stranded cetaceans, demonstrating that welfare science can, and must, be integrated alongside conservation biology at cetacean strandings events. These indicators should be investigated at future stranding events, and where data is available, retrospective analysis from past strandings conducted, to assess their feasibility, and to enable an assessment of their reliability and validity to inform decision-making. In this way stranded cetacean state can be unambiguously assessed, and measurable objectives established for conservation goals, both regionally and globally, that incorporate animal welfare.

## Data Availability

The processed datasets supporting this article have been uploaded as part of the electronic supplementary saterial [106].
